# The Effects of Low Concentrations of Nisin on Biofilm Formation by *Staphylococcus aureus* Isolated from Dairy Cattle †

**DOI:** 10.3390/pathogens14060566

**Published:** 2025-06-05

**Authors:** Edyta Kaczorek-Łukowska, Paweł Foksiński, Joanna Małaczewska, Roman Wójcik, Natalia Szyryńska

**Affiliations:** 1 Department of Microbiology and Clinical Immunology, Faculty of Veterinary Medicine, University of Warmia and Mazury in Olsztyn, Oczapowskiego 13, 10-719 Olsztyn, Poland; pawel.foksinski@uwm.edu.pl (P.F.); joanna.malaczewska@uwm.edu.pl (J.M.); brandy@uwm.edu.pl (R.W.); 2 Department of Histology and Embryology, Faculty of Veterinary Medicine, University of Warmia and Mazury in Olsztyn, Oczapowskiego 13, 10-719 Olsztyn, Poland; natalia.skiepko@uwm.edu.pl

**Keywords:** bacteriocins, nisin, virulence, staphylococci, *icaD*

## Abstract

*Staphylococcus aureus* is one of the aetiological agents of mastitis in dairy cattle. Their biofilms are relevant for human and veterinary medicine. It has been shown that some antibiotics at low concentrations can stimulate the production of biofilms, but there is little information on the effects of low concentrations of nisin, which is considered a therapeutic agent and has been added to food products for years as a biopreservative. In our study, we used *Staphylococcus aureus* strains (*n* = 28) isolated from dairy cattle. The MIC of nisin were determined using the broth microdilution method. Based on the minimum inhibitory concentration (MIC) results, the following concentrations were selected for further analyses: nisin at 39, 19, 9 IU/mL; nisin in combination with tetracycline at 39 IU/mL + 0.06 μg/mL, 18 IU/mL + 0.06 μg/mL, and 9 IU/mL + 0.06 μg/mL; and tetracycline alone at 0.06 μg/mL. The biofilm-forming capacity was determined via crystal violet staining in 96-well plates, *icaD* gene expression was determined using the 2−ΔΔCt method, and microscopic evaluation was carried out using scanning electron microscopy. **Results**: The MICs were 156 IU/mL (46%) and 312 IU/mL (43%) for most strains. Due to large statistical deviations, there were no statistically significant changes in the biofilm-forming capacity or *icaD* gene expression despite a visible increasing trend. Despite the absence of statistically significant differences, it was observed that for all concentrations analysed biofilm formation was noticeably greater for both nisin alone and for tetracycline and its mixtures than for untreated cells. **Conclusions**: In our opinion, the effects of nisin, especially at low concentrations, on biofilm structure show a certain worrying trend that may pose a future threat.

## 1. Introduction

*Staphylococcus aureus* (*S. aureus*) is an important pathogen in both human and veterinary medicine [1]. This bacterium plays a special role as one of the main aetiological agents of mastitis in cattle and is one of the reasons for large economic losses in the dairy industry due to reduced milk quality and production [2]. This bacterium is mainly associated with infections that are hard-to-treat, and therefore often persistent due to both its antibiotic resistance and numerous virulence mechanisms, which it acquires as a result of its genetic flexibility [3]. One of these mechanisms that relates to both antibiotic resistance and virulence is the ability to produce a biofilm. Biofilms are largely responsible for the recurrence of infections since their multilevel structure allow survival in unfavourable conditions and protects pathogens from the application of antibiotics [4]. It is estimated that bacteria capable of forming a biofilm structure are up to one thousand times more resistant to a specific active substance compared to the same pathogens growing in planktonic form, preventing targeted therapies from guaranteeing therapeutic success [4,5]. Moreover, biofilms contribute significantly to the spread of drug resistance as they enable both horizontal and vertical transfer of genes associated with this phenomenon through their complex multicellular structure. Although the resistance to antibiotics of bacteria in biofilm structures is well known, in the case of bacteriocins the use of antiseptic disinfectants or other substances in preventive or therapeutic procedures for *S. aureus* has been relatively poorly studied. Bacteriocins are used in the food industry as biopreservatives due to their natural origin, low toxicity and narrow spectrum of action; however, they are being increasingly explored for clinical use in both animals and humans [6,7]. One such bacteriocin is nisin, produced by *Lactococcus lactis* subs. *Lactis*, which is active against multiple Gram-positive bacteria and is currently registered as a biopreservative (E 234) in the European Union (EU) under Regulation (EC) 1333/2008 for use in several food categories [8]. In addition, nisin is being considered for medical use in the treatment of various types of infections caused by multidrug-resistant strains, oral infections, immunomodulation or anticancer therapies [6]. However, it is increasingly being reported that bacteria are acquiring resistance mechanisms against nisin. To date, this phenomenon has been observed in *Lactobacillus casei*, *Streptococcus thermophilus*, *Pediococcus acidilactici*, *Streptococcus bovis*, *Listeria monocytogenes*, *Bacillus cereus*, *Staphylococcus aureus* and *Clostridium botulinum* [9]. Worryingly, in 2017 the European Food Safety Authority (EFSA) Panel on Food Additives and Nutrient Sources Added to Food (ANS) issued recommendations to analyse the potential of inducing antimicrobial resistance (AMR) to nisin in pathogenic bacteria through its use as a food additive [10]. In our view, this is a clear indication that this problem will increase as a result of the accumulation of food industry waste, and the possible introduction of nisin into the medical market may only intensify this problem, which is why this phenomenon should be monitored. In light of this information, we tested how low nisin concentrations might affect the biofilm-forming ability of *S. aureus* strains obtained from dairy cattle.

## 2. Materials and Methods

### 2.1. Reagent Preparation for Biofilm, Gene Expression and SEM Analysis

Nisin (2.5%) from *Lactococcus lactis*, with a potency of 1,027,000 IU/g, was purchased from Sigma Aldrich, Taufkirchen, Germany. To obtain a working stock solution (250 IU/mL), 100 mg of nisin was dissolved in 411 mL of ultrapure water, then the solution was filtered (0.22 μm filter, Millipore, Burlington, MA, USA). Tetracycline hydrochloride (with a potency of 897.33 μg/mg) was purchased from Sigma Aldrich, Taufkirchen, Germany. To obtain the working stock (2560 μg/mL), 250 mg was dissolved in 88 mL of ultrapure water. Working dilutions were prepared from the stock in tryptic soy broth TSB broth (Oxoid, Basingstoke, UK) with the addition of 1% glucose:

- nisin: 39, 19, and 9 IU/mL
- tetracycline 0.06 μg/mL
- mixture of nisin and tetracycline: 39 IU/mL + 0.06 μg/mL, 18 IU/mL + 0.06 μg/mL, and 9 IU/mL + 0.06 μg/mL.

### 2.2. Strains

*S. aureus* strains (*n* = 28) isolated from dairy cattle with subclinical mastitis between 2016 and 2019 from northeastern Poland were used for the study. To avoid epidemiologically related strains, only one strain per farm was collected. Detailed information about the strains and their identification can be found in the previously published article [3].

### 2.3. Minimum Inhibitory Concentration Analysis for Nisin

Minimum inhibitory concentrations were determined using the broth microdilution method in accordance with the recommendations of the Clinical and Laboratory Standards Institute [11]. The test was performed on Cation Adjusted Mueller Hinton Broth (CAMHB) in 96-well plates. Each strain was validated at 5 × 10^4^ CFU/well in accordance with CLSI. Samples were incubated at 35 ± 2 °C for 16–20 h. The MIC for each strain was determined by unaided visual inspection. Nisin (Sigma Aldrich, Taufkirchen, Germany) was tested at a concentration range of 2, 4–1250 IU/mL. All the antimicrobial agents were filtered through a 0.22-μm pore size Millipore filter (Merck Millipore, Billerica, MA, USA).

### 2.4. Biofilm Formation Assay

The experiment was performed using polystyrene microtitre plates with flat bottoms based on the techniques described by [12] with slight modifications. The concentration of the strains in all samples was 5.6 × 10^8^ cells/mL. For each strain (*n* = 28), the same test panel was placed on the plate. The control group (untreated strains in TSB broth with 1% glucose) and treated groups: nisin alone (39, 19, and 9 IU/mL), nisin in combination with tetracycline (39 IU/mL + 0.06 μg/mL, 18 IU/mL + 0.06 μg/mL, 9 IU/mL + 0.06 μg/mL), and tetracycline alone (0.06 μg/mL). All assays were performed in triplicate with 8 replicates for each strain with negative control (TSB broth alone with 1% glucose). Samples were incubated under aerobic conditions at 37 °C for 48 and 72 h. Next, the plates were washed three times with sterile phosphate-buffered saline (PBS) (MP Biomedicals Europe, Warsaw, Poland). The plates were left to dry inverted at ambient temperature for 1 h, after which they were stained with 200 μL of 0.2% aqueous crystal violet solution (Sigma Aldrich, Taufkirchen, Germany) for 15 min. The plates were then washed three times with sterile PBS to remove the excess dye. The crystal violet bound to the biofilms was then extracted using a 200 μL mixture of 80% ethyl alcohol and 20% acetone. The results were read at 595 nm using an ELISA plate reader (Sunrise Absorbance Reader, Tecan, Austria).

### 2.5. Gene Expression of the icaD Gene

For this analysis, only 5 strains (ID 360,544,228,312,522) in which increased biofilm production was observed in the presence of low concentrations of nisin were selected. All selected strains were non- or weak biofilm producers after 24 h. The analysis was focused on the *icaD* gene, as its presence was the only one that was confirmed among the analysed strains [3]. The number of bacterial cells was standardised to 4.5 × 10^8^ cells/mL, and subsequently 25 µL of this suspension was added to 975 µL of TSB broth (Oxoid, Basingstoke, UK), supplemented with 1% glucose and an appropriate concentration of nisin alone (39, 18, 9 IU/mL), tetracycline (0.06 µg/mL) or a mixture of both (39 N + T, 18 N + T, 9 N + T) on 24-well plates. Each assay was performed with a control sample (untreated strain in TSB broth with 1% glucose) and a negative sample (pure broth). The samples were incubated at 37 °C under aerobic conditions for 8, 12, 24 or 48 h. After incubation, the RNA was isolated with a commercial RNA Mini Kit (A&A Biotechnology, Gdynia, Poland) according to the manufacturer’s instructions. The concentrations of eluted RNA were measured with a NanoDrop 2000 spectrophotometer (Thermo Fisher Scientific, Waltham, MA, USA) and the samples were stored at −80 °C until further analysis. Reverse transcription was carried out with a High-Capacity cDNA Reverse Transcription Kit (Life Technologies, Waltham, MA, USA) according to the manufacturer’s instructions. All RNA samples were standardised to 0.3 μg per sample for the synthesis of complementary DNA (cDNA). The expression of the gene encoding the *icaD* gene was determined using real-time PCR. The reaction mixture for all analysed genes had the following composition: 10 μL of Power SYBR^®^ Green PCR Master Mix (Life Technologies, Waltham, MA, USA), 0.6 μL of each 10 μM primer, 6.8 μL of RNase-free water, and 2 μL of cDNA. All detailed information about the primer sequences is summarised in Table 1. The reaction was carried out under the following conditions: polymerase activation at 95 °C for 10 min, followed by 40 three-stage cycles of denaturation at 95 °C for 30 min, primer annealing at 53 °C for 30 s and chain elongation at 53 °C for 30 s. All assays were performed in triplicate for each strain The relative expression of each gene was calculated using the 2−ΔΔCt method normalised to efficiency corrections, expression levels of reference genes encoding cell division proteins (*ftsz*) and the control group (untreated strains in TSB broth with 1% glucose).

### 2.6. Scanning Electron Microscopy

For this analysis only 2 strains (ID 544,360) in which increased biofilm production was observed in the presence of low concentrations of nisin alone or in combination with tetracycline were selected. The experiments were performed using glass coverslips in 6-well plates. The number of bacterial cells was standardised to 4.5 × 10^8^ cells/mL, then 100 µL of this suspension was added to 3900 µL of TSB broth (Oxoid, Basingstoke, UK) supplemented with 1% glucose and an appropriate concentration of nisin alone (39, 18, 9 IU/mL), tetracycline (0.06 µg/mL) or a mixture of both (39 N + T, 18 N + T, (N + T) on 24-well plates. Each assay was performed with a control sample (untreated strain in TSB broth with 1% glucose) and a negative sample (pure broth). The samples were incubated at 37 °C under aerobic conditions for 48 h. Then, the bacterial biofilms on coverslips were immersion-fixed in a mixture of 2.5% glutaraldehyde and 1% paraformaldehyde in 0.2 M phosphate buffer (pH 7.4) for 2 h at 4 °C. They were washed three times in 0.2 M phosphate buffer (pH 7.4), dehydrated in ethanol (30, 50, 70, 90, 95, 100%, 15 min each), dried in a critical point dryer (Autosamdri^®^-931, Tousimis, Rockville, MD, USA) and chromium-coated (10 nm) using a sputter coater (Quorum Q150T ES, Quorum Technologies, Laughton, UK). Thereafter, the biofilms were imaged under a field-emission scanning electron microscope (Gemini 450, Carl Zeiss, Oberkochen, Germany) at an acceleration voltage of 1.3 kV using an Everhart–Thornley SE detector.

### 2.7. Statistical Analysis

All results are expressed as mean ± standard deviation (SD). The data were subjected to a nonparametric test and the Kruskal–Wallis test was used to determine differences between the control and study groups. Statistical evaluation of the results was performed using GraphPad Prism 7 software (GraphPad Software, San Diego, CA, USA).

## 3. Results

### 3.1. Minimum Inhibitory Concentration Analysis for Nisin

**Figure 1:**
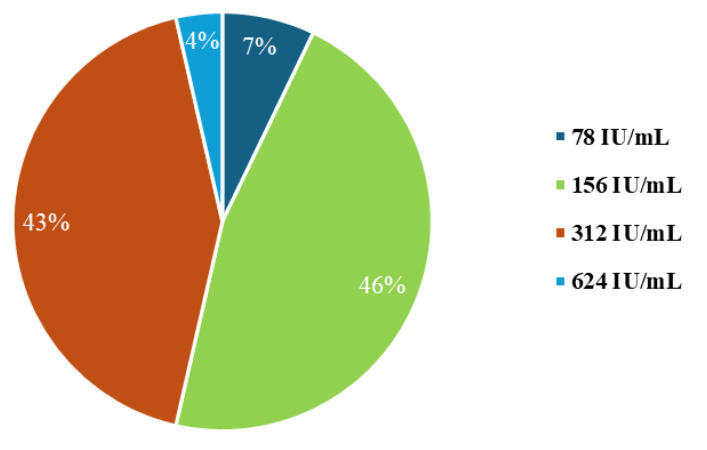
Distribution of nisin MIC values for *S. aureus* strains (*n* = 28).

The MIC values were 156 IU/mL (46%) and 312 IU/mL (43%) for most strains. The other concentrations of 78 IU/mL and 624 IU/mL were less than 10% of the strains analysed (Figure 1).

### 3.2. Results of Biofilm-Forming Ability

**Figure 2:**
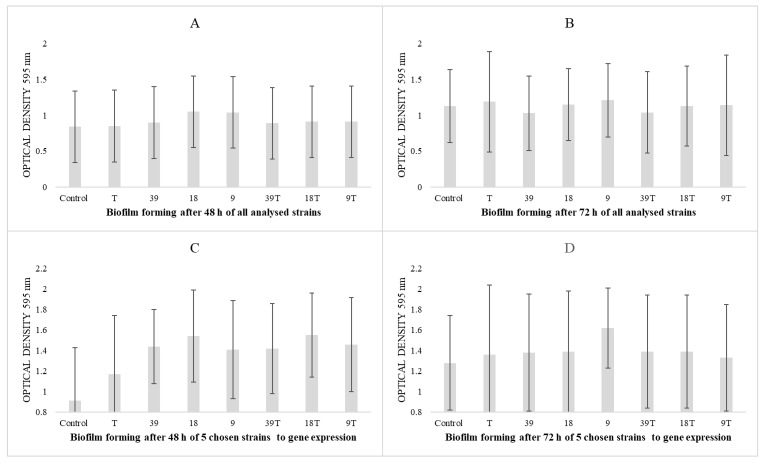
Results of biofilm forming ability against analysed concentrations of nisin, tetracycline and their mixtures. Concentration of nisin (39, 18, 9 IU/mL), tetracycline (T 0.06 ug/mL) and mixtures of nisin and tetracycline 0.06 ug/mL (39 T, 18 T, 9 T), Control-group strains cultured in TSB with 1% glucosepp. Samples were incubated under aerobic conditions at 37 °C for 48 and 72 h. (**A**)—results for all *S. aureus* strains (*n* = 28) after 48 h, (**B**)—results for all *S. aureus* strains (*n* = 28) after 72 h, (**C**)—results for *S. aureus* strains (*n* = 5) selected for gene expression after 48 h, (**D**)—results for *S. aureus* strains (*n* = 5) selected for gene expression after 72 h.

Due to large statistical deviations, there were no statistically significant changes in the biofilm-forming capacity assays despite a visible increasing trend for all 28 strains (Figure 2A,B) or selected strains for gene expression (*n* = 5) (Figure 2C,D) at both 48 and 72 h.

### 3.3. Expression of the icaD Gene in 5 Selected Strains

**Figure 3:**
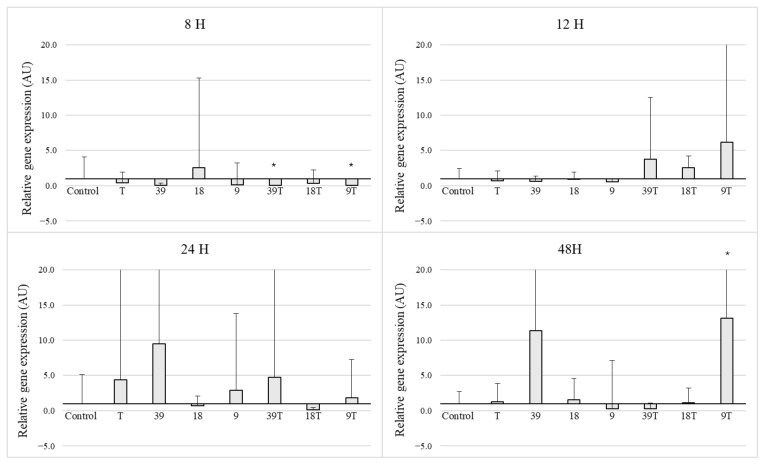
Results of gene expression of *icaD* gene in *S. aureus* (*n* = 5). Concentrations of nisin (39, 18, 9 IU/mL), tetracycline (T 0.06 ug/mL) and mixtures of nisin and tetracycline 0.06 ug/mL (39 T, 18 T, 9 T), Control-untreated strains cultured in TSB with 1% glucose. The mean relative expression values above 1 (black line) in analysed groups indicate higher gene expression in comparison with the control group. Results for incubation after 8 h, 12 h, 24 h and 48 h. All data expressed as means ± SD (standard deviation) for analysed strains (*n* = 5) in arbituary units (AU). Asterisks refer to statistically significant differences between control and treated strains: * *p* < 0.05.

Due to large statistical deviations in most cases among the analysis times of 8, 12, 24 and 48 h, no statistically significant changes in *icaD* gene expression were observed despite a visible increasing trend. The only exceptions were: inhibition of expression after 8 h for concentrations 39 T and 9 T and increases in *icaD* gene expression after 48 h for concentration 9 T (Figure 3).

### 3.4. Scanning Electron Microscopy

**Figure 4:**
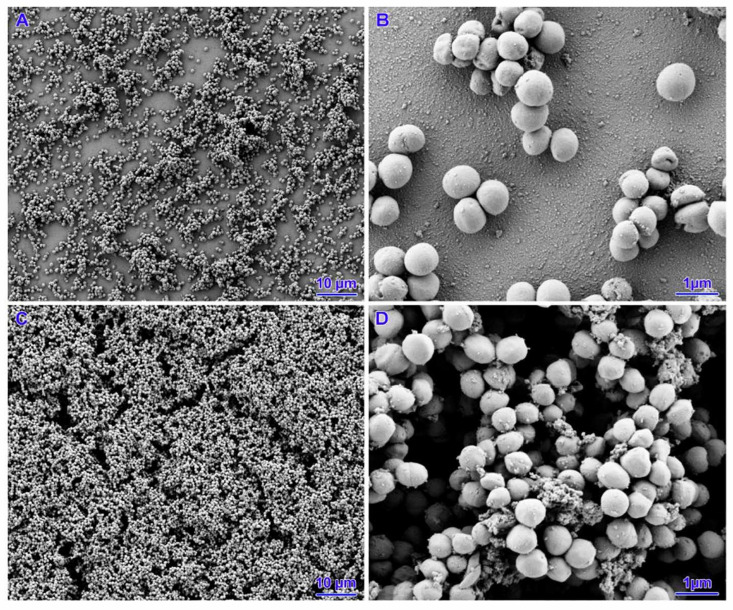
Results of biofilm formation after 48 h. (**A**,**B**)—untreated strain. (**C**,**D**)—nisin 39 IU/mL.

**Figure 5:**
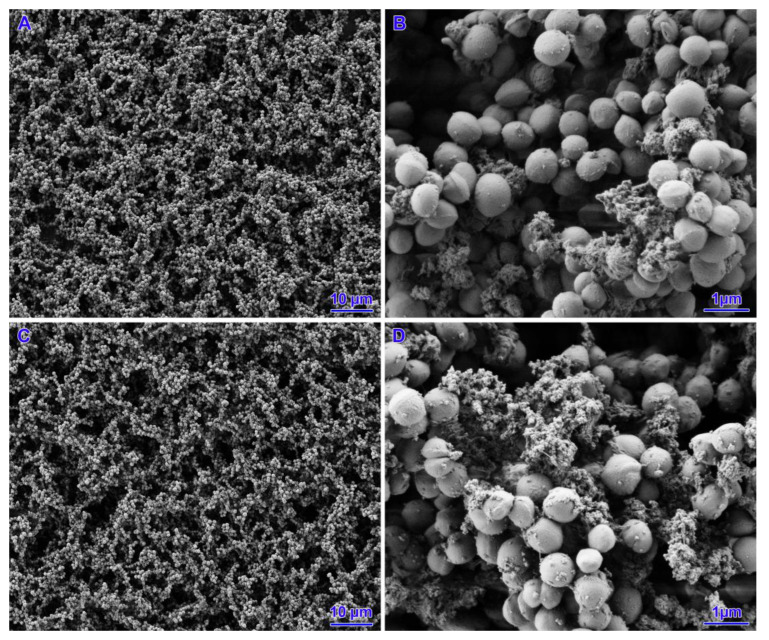
Results of biofilm formation after 48 h. (**A**,**B**)—nisin 39 IU/mL and tetracycline 0.06 ug/mL. (**C**,**D**)—nisin 18 IU/mL and tetracycline 0.06 ug/mL.

**Figure 6:**
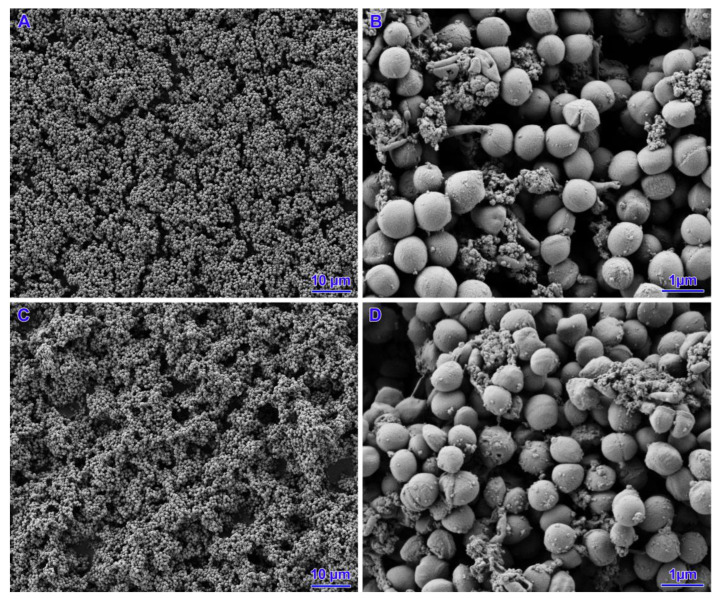
Results of biofilm formation after 48 h. (**A**,**B**)—nisin 18 IU/mL. (**C**,**D**)—nisin 9 IU/m.

**Figure 7:**
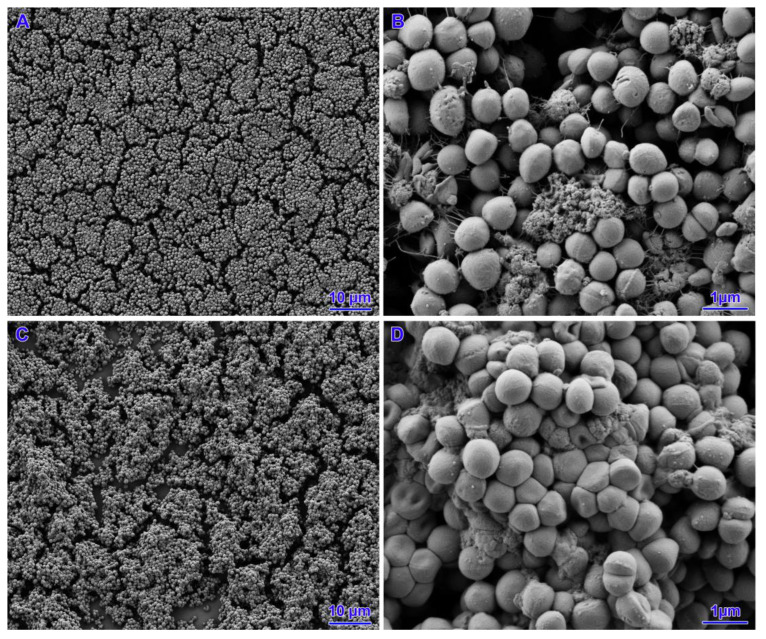
Results of biofilm formation after 48 h. (**A**,**B**)—tetracycline 0.06 ug/mL. (**C**,**D**)—nisin 9 IU/mL and tetracycline 0.06 ug/m.

Two strains (out of five strains subjected to *icaD* gene expression) with poor biofilm formation ability were selected for SEM analysis. In the case of the untreated strains, a small number of cells were observed that did not cover the entire surface of the slide. The presence of small aggregates of cells with single junctions into a monolayer and a three-dimensional structure was confirmed. For all concentrations analysed, both nisin alone, tetracycline and their mixtures, coverage of the entire slide surface, an increase in the number of bacterial cells and the appearance of exopolysaccharides (EPS) were observed. The biofilms had a mature three-dimensional structure and were characterised by integrity. However, there was no change in the morphology of the staphylococcal cell structure (Figure 4, Figure 5, Figure 6 and Figure 7).

## 4. Discussion

Bacterial biofilms are structures commonly found in the natural environment (plant roots, rocks or mammalian teeth). According to some authors, the ability to form this structure is one of the most flexible characteristics of microorganisms [15]. An increasing number of bacteria are found to have the ability to form biofilms. This is a particularly dangerous development in the case of pathogens, as it is becoming a crucial virulence factor. *Staphylococcus* spp., a well-known biofilm producer, can cause severe and recurring infections in both humans and animals [5]. To make matters worse, in addition to colonising the organism, in this form they can also contaminate both medical devices (e.g., catheters) and dairy equipment (milking parlours, milk tanks), which can infect large groups of animals [15,16,17]. The formation of this structure is aimed at the adaptation of pathogens to unfavourable conditions such as low nutrient levels, UV radiation, temperature changes or biological and chemical influences [5]. As mentioned earlier, there is little information on biofilm resistance to bacteriocins, natural compounds or disinfectants. Most publications have focused on the effect of subclinical doses of antibiotics on resistance, as well as their effects on bacterial biofilms. In this case, it has been shown that some of these compounds can stimulate biofilm synthesis at low concentrations, but there is increasing discussion concerning how other factors can affect this structure [8,18].

Nisin is one of the most popular bacteriocins and is widely used in the food industry; it has the potential to become a therapeutic agent so it is essential to determine how its low concentrations affect pathogenic bacteria. In particular, subinhibitory concentrations (sub-MICs) can occur with antibiotic therapy, contributing to the development of antibiotic resistance. In other words, low concentrations of the active substance administered allow some bacteria to survive and multiply at a slower rate [19]. Sub-MICs occur in both human and veterinary medicine as a result of inadequate treatment regimens, poor drug penetration, inappropriate dosing, drug–drug interactions or resistance of the bacteria to the active substance used. At sub-MIC concentrations, *S. aureus* can induce the formation of small colony variants (SCVs) and changes in cell morphology as well as influence the expression of various virulence factors, including biofilm-forming ability [20]. Given that nisin has been widely used in the food industry for years and resistance has already been observed, it cannot be excluded that its sub-MIC values have the same effect as antibiotics on *S. aureus*, which is a very important pathogen for both humans and animals [9].

In our study, no statistically significant changes in biofilm-forming capacity were observed, but an increasing trend was observed for both nisin alone and for their mixtures with tetracycline. Our results are different from those of other research teams. In most cases, nisin at MICs or lower concentrations have inhibited biofilms on the tested bacteria *S. aureus*, *Streptrococus uberis*, *Pseudomonas aeruginosa*, and *Listeria monocytogens* [21,22,23]. Most of these studies were carried out on a small number of strains or reference strains, making the standard deviations small. These types of results are very good for providing an overview of the situation, identifying a problem and flagging its existence. In our case, we collected results for 28 field strains and selected five for gene expression, which means that the standard deviations are large, but we believe this more accurately reflects the true situation in the environment. Nonetheless, results similar to ours were observed by [24,25], where an increase in biofilm production by *S. aureus* was also observed for sub-MIC values. In both of these studies, it was observed that nisin in concentrations of one-eighth and even one-quarter of the MIC stimulated biofilm growth in *Staphylococcus aureus*. This shows that, as with antibiotics, the effect of sub-MIC concentrations of nisin is quite controversial. This can also be seen in our study, where although no statistically significant differences in biofilm production were shown at 48 and 72 h for all strains analysed, a trend towards increased biofilm production can be seen among the majority of concentrations analysed. Moreover, SEM analysis of two of our selected strains revealed a significant increase in biofilm production. Depending on the strain, geographical latitude, type of nisin (potency and purity), time and site of isolation, antibiotics may induce or decrease biofilm formation, and therefore may decrease or increase the in vitro expression of virulence-related genes [19,20]. One of the mechanisms of biofilm formation is the accumulation of bacterial cells to produce a multilayered structure of cells surrounded by a mucous substance. The operon *ica* is key to the synthesis of this structure through the synthesis of glucosamine polymer (PIA) [26], which is responsible for adhesion and, ultimately, for the formation of biofilms of bacteria in the *Staphylococcus* genus [14]. In the case of our strains from the *ica* operon, only the *icaD* gene was confirmed among the analysed strains and subjected to a gene expression study [3]. As with the biofilm-forming ability, no statistically significant results were obtained in most cases but an increase in this gene could be observed at different times and concentrations of nisin, tetracycline and their combinations. These results are partly consistent with data from other authors. In our study, peaks in *icaD* gene expression were observed at different times depending on the tested substances. Most occurred at 8, 24 and 48 h. According to some authors, the peak of *icaD* gene expression occurred between 3 and 8 h, after which it decreased [14,27]. This suggests that this gene should be most active during the first phase of biofilm formation and should then be replaced by other genes. In our research, the mechanism was variable. This finding suggests that, in addition to the *ica* operon, other mechanisms not examined by us such as the binding factors lamimin (*eno*), elastin (*ebps*), fibrinogen binding protein (*fib*), the accumulation-associated protein (*Aap*), and the accumulation of extracellular DNA or major cell wall autolysin *atlE*, can be activated by sub-MIC concentrations of nisin [14,28]. Another reason for this variability in the expression of this gene over time is that several regulators are involved in the synthesis of PIA, including *icaR*. As reported by [29], if this gene is removed, PIA production is upregulated. In addition, as reported by [30], some antibiotics from the aminoglycoside group, e.g., gentamicin, can cause *icaR* to bind to DNA, resulting in the induction of biofilm formation. It is possible that nisin at sub-MIC concentrations has a similar mechanism, but further research in this direction should be performed.

As previously mentioned, nisin is widely used in the food industry as a biopreservative and is considered therapeutic for both humans and animals. This means that its concentration in the environment will continue to increase and that bacterial resistance to it will likely increase as a result of food waste, inappropriate disposal, untargeted therapies or preventative measures. Moreover, low concentrations of antibiotics including tetracycline, which can stimulate biofilm formation in various bacteria, circulate in the environment. We do not know if there is a correlation between low concentrations of nisin and other substances encountered in the environment [18].

In our opinion, the effects of nisin, especially at low concentrations, on bacteria in planktonic form as well as on the biofilm structure should be continuously monitored. These results are ambiguous but they show a certain worrying trend that may suggest a future threat. This article is a revised and expanded version of a paper entitled “Is nisine future salvation or a potential threat? Results of a study of the effect of low concentrations of nisin on ability on biofilm formation by Staphylococcus aureus isolated from dairy cattle”, which was presented at XVII Congress of the Polish Society of Veterinary Sciences: “Sanitas animalium pro salute homini”, in Poland, Olsztyn 19–21 September 2024 [31].

## Figures and Tables

**Table 1:** Sequence of primers used in the gene expression study.

Primer Name	Primer Sequence (5′–3′)	Reference
*ftsz*	ATCCAAATCGGTGAAAAATTAACAC CCATGTCTGCACCTTGGATTG	[13]
*icaD*	TCAAGCCCAGACAGAGGGAATA ACACGATATAGCGATAAGTGCTGTTT	[14]

## Data Availability

The datasets used and/or analysed during the current study are available from the corresponding author on reasonable request.

## Abbreviations

AMR	antimicrobial resistance
AU	arbituary units
CAMHB	Cation Adjusted Mueller Hinton Broth
MIC	minimum inhibitory concentration
PBS	phosphate-buffered saline
EPS	exopolysaccharides
TSB	tryptic soy broth
kV	kilovolt
SEM	scanning electron microscopy
EPS	exopolysaccharide
